# Apolipoprotein D acts as a chaperone of Aβ peptide delaying its assembly into amyloid aggregates

**DOI:** 10.1007/s00018-026-06339-2

**Published:** 2026-07-09

**Authors:** Jorge Mondejar-Duran, Antonio Díaz-Quintana, Maria D. Ganfornina, Diego Sanchez

**Affiliations:** 1 Instituto de Biomedicina y Genética Molecular, Universidad de Valladolid-CSIC, Valladolid, 47003 Spain; 2 Instituto de Investigaciones Químicas, Centro de Investigaciones Científicas Isla de La Cartuja, Universidad de Sevilla-CSIC, Seville, Spain

**Keywords:** Amyloid-β, Alzheimer’s disease, Thioflavin T, Molecular dynamics, High-performance docking, Tryptophan fluorescence titration, Anti-aggregation

## Abstract

The accumulation of amyloid-β (Aβ) peptides is at the root of Alzheimer’s disease (AD), where oligomeric forms of Aβ disrupt brain physiology before any sign of dementia. Intense research has been directed to extrinsic molecules that modulate Aβ aggregation. However, more knowledge is needed about endogenous mechanisms maintaining Aβ homeostasis in a healthy brain. The Lipocalin Apolipoprotein D (ApoD) displays neuroprotective functions, many of which rely on managing lipids in cell membranes or lipoprotein particles. ApoD is upregulated in the aging brain and AD and is present in amyloid-β plaques. However, the functional relationship of ApoD with Aβ peptides aggregation needs to be studied. Using aggregation and ligand binding assays guided by molecular dynamics modeling, in this work we demonstrate that human ApoD is able to interact with Aβ, both in monomeric or aggregated states. Multiple binding sites in ApoD, including its hydrophobic ligand-binding pocket, are demonstrated between ApoD and Aβ by fluorescence titration, and these diverse interactions delay the aggregation process and reduce the number of large aggregates. Modeling the dynamics of interactions in sub-stoichiometric ApoD/Aβ ratios predicts that ApoD decreases the probability of switching to the β-stranded structure required for Aβ oligomerization. Our findings reveal Aβ as a novel ligand category for ApoD and therefore place this Lipocalin as an important element of the endogenous control of Aβ. ApoD is able to chaperon Aβ monomers from their origin in cellular membranes, preventing their progression towards amyloid plaques.

**Graphical abstract:**

**Figure:**
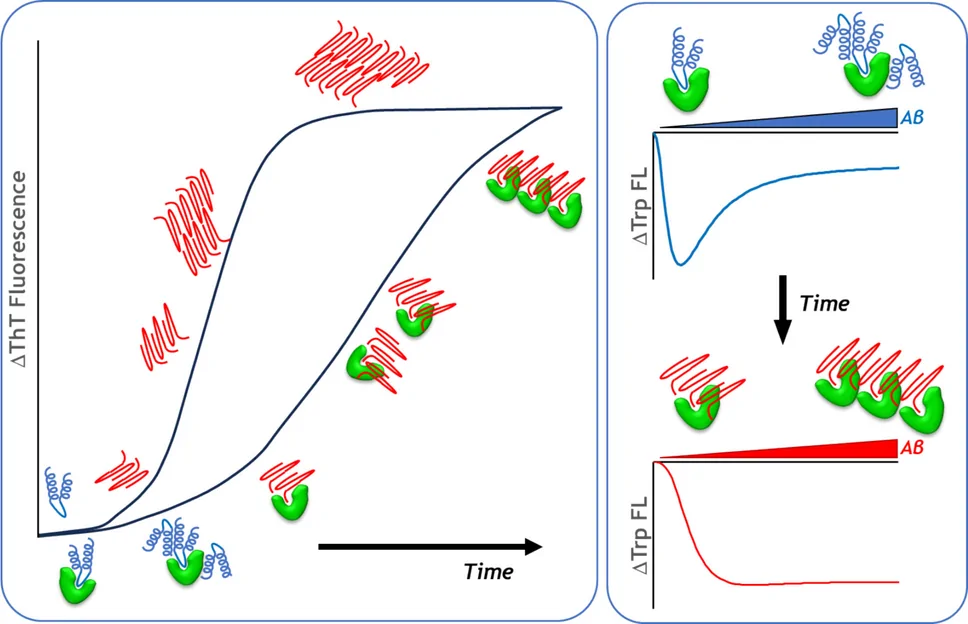

**Supplementary Information:**

The online version contains supplementary material available at https://doi.org/10.1007/s00018-026-06339-2.

## Introduction

Understanding how the brain physiologically regulates the amyloid beta peptide (Aβ) has long driven efforts to decipher the pathogenesis of Alzheimer’s disease (AD), since the failure to maintain tolerable extracellular levels of this peptide is a critical factor to prevent the onset of this devastating disorder affecting approximately 40–50 million individuals worldwide [1, 2]. When Aβ levels exceed the physiological threshold [3, 4] the peptide undergoes aggregation losing solubility and initiating a cascade of neurotoxic events [5] that include disruption of synaptic function, cellular stress-induced damage and localized neuroinflammation, ultimately leading to neuronal death and progressive neurodegeneration [6].

Aβ is generated by a particular form of processing the amyloid precursor protein (APP) [7, 8], a protein whose physiological function remains under debate [9, 10]. This process involves sequential cleavage by β-secretase (BACE1) and γ-secretase within specific liquid-ordered membrane domains (lipid rafts) of the endosomal vesicular trafficking system [10]. Various forms of APP precursor cleavage gives rise mostly to peptides with 40 or 42 amino acids (Aβ_1–40_ and Aβ_1–42_), but also to peptides Aβ_17–40_ or Aβ_17–42_ that show different aggregation and toxicity properties [8]. All versions of Aβ form highly toxic oligomers if the peptides released to the extracellular space are not efficiently cleared or sequestered into amyloid plaques [11]. Therefore, controlling the production and accumulation of low-molecular weight Aβ aggregates is essential for preserving brain homeostasis and neuronal integrity.

Apolipoprotein D (ApoD) is a member of the Lipocalin family [12] expressed in the nervous system by myelinating glia, astrocytes and pericytes in a tightly regulated manner, with specific conditions triggering its expression [reviewed by [13]. ApoD is associated with lipoprotein particles and cellular membranes, where it interacts with particular subtypes of lipid rafts [14]. ApoD traffic from the plasma membrane to the endolysosomal compartment is promoted by oxidative stress [15] and it is also exported as a peripheral membrane protein of extracellular vesicles [16]. Our systematic review of ApoD function [13] led us to propose a unique biochemical role in lipid management of cellular membranes and extracellular structures, that accounts for its wide range of functions at cellular and organismal level, from myelin compaction to protection against oxidative stress, neuroinflammation, and neurodegeneration (reviewed by [17]).

Experimental evidence in model organisms of neurodegenerative diseases demonstrates that ApoD and its invertebrate homologues exert neuroprotective functions in pathologies of different etiology [18–20]. Of relevance to the present work, ApoD has been demonstrated to modulate amyloid pathology in a mouse model of AD [21]: The lack of ApoD in APP/PS1 mice causes an increase in amyloid plaque deposition, an elevation of BACE1 protein and an intensified microglial activation, while ectopic expression of ApoD in neurons reduce their amyloid plaque load.

An abundant literature links ApoD over-expression with AD [1998–2020 reviewed by [13, 22–25], and histopathological analyses identify ApoD location in Aβ compact plaques and reactive astrocytes surrounding them [26, 27]. This location in AD patients and the effects in plaque load in the AD mouse model [21] are compatible with ApoD being involved in modulating plaque formation and architecture and the concomitant glial responses.

However, Aβ plaques are complex structures composed of approximately 2000 proteins [28–30]. Besides ApoD, another member of the Lipocalin family, Prostaglandin D Synthase (L-PGDS), and several apolipoproteins (ApoE, ApoA-I and ApoJ) have been detected in the vicinity of human Aβ plaques, with ApoE colocalizing with ApoD in certain plaques [26]. These data suggest synergistic or overlapping functions for these lipid binding proteins in AD pathology [31].

It is therefore mandatory to test whether human ApoD has an active role conditioning Aβ dynamics in the extracellular space, before we can propose causal relationships to the reported correlations. The null hypothesis is that ApoD is a protein passively trapped in amyloid plaques, a situation that would be favored in the ApoD overexpressing conditions taking place in both physiological and pathological aging of the human brain.

The goal of this work is to investigate if ApoD can interact with Aβ and alter its aggregation dynamics. We use biochemical and computational approaches to characterize this potential role of ApoD as an active Aβ modulator. Our study validates our working hypothesis and places ApoD as a neuroprotective agent in AD, with biochemical properties that enable ApoD to bind Aβ peptides as soon as they are produced in intracellular membranes and to chaperon them until they form part of amyloid plaques.

## Methods

### Proteins, peptides and reagents

Thioflavin T (ThT, Sigma-Aldrich) was resuspended in methanol to a stock concentration of 60 mM and further diluted in phosphate-buffered saline (PBS) (intermediate 60 µM stock) just before use.

Monomeric Aβ1–42 (rPeptide or Bachem), Aβ1–28 and Aβ35–42 (Bachem) were solubilized in hexafluoroisopropanol (HFIP, Sigma-Aldrich), aliquoted in Snap Cap Low Retention Microcentrifuge tubes (Thermo), dried for 15 min at room temperature (RT) in a SpeedVac system, and stored at −80ºC in closed bags with desiccant. Immediately prior to use, peptides were resuspended in 1% NH4OH (1–2 mM stock concentration) and incubated on ice for 10 min. These steps are critical to ensure initiation of aggregation assays in monomeric state.

We used ApoD purified in its native form from breast cystic fluid (ApoD^BCF^) [32], or a recombinant bacterial form (ApoD^Bact^, Prospec). Other proteins used to test specificity were trypsin inhibitor (TI, Pierce™), and Alpha-1-acid glycoprotein (AGP) and Alpha−1-microglobulin (A1M) (kindly provided by Dr. Bo Åkerström).

### Thioflavin T aggregation and disaggregation assays

Aggregation kinetic was monitored by adding monomeric Aβ_1–42_ to the protein of interest in PBS (or PBS alone) with 20 µM ThT [33]. Samples (100 µl) were dispensed in black/clear flat bottom 96-well plates (Falcon). We performed in situ kinetic or endpoint assays to measure ThT fluorescence (Ex 440/450 ± 10 nm; Em 485/490 ± 10 nm) in a Cytation 5 plate reader (CYT5MFV; BioTek). For spontaneous Aβ aggregation, ThT fluorescence was recorded every 15 min over 20–40 h at 37 °C without shaking. ThT fluorescence, after subtraction of ThT signal in PBS, was fitted to a four parameters sigmoidal function [34] from which *t*_lag_ (time to the beginning of fibril elongation phase) and *k*_app_ (apparent elongation rate) were calculated. In endpoint experiments of accelerated Aβ aggregation ThT fluorescence was recorded first every 30 min over 3–24 h at room temperature (RT) under orbital shaking (800 rpm), and a 3 h interval was chosen for endpoint measures in optimization or specificity experiments. Optimization included excitation-emission parameters, incubation temperature and protein/peptide concentrations (Supplementary Fig. S1). After each aggregation assay, images were taken using the Cytation 5 imaging module with the GFP filter set (Ex/Em 469/525 nm) using 4 × and 10 × objectives. Images were acquired under the same illumination and exposure time conditions and analyzed using the FiJi program [35].

For disaggregation assays, Aβ_1–42_ monomers resuspended in NH₄OH were allowed to spontaneously aggregate for over 3 months at −80ºC and then mixed ThT and 50 nM ApoD^BCF^. ThT fluorescence was monitored for 24 h. As a positive control for disaggregation, Aβ_1–42_ aggregates without ApoD were subjected to sonication (3 min) prior to ThT addition. Control fluorescence values of ThT alone were recorded in every aggregation and disaggregation experiment.

### Protein immunoblots

ApoD^BCF^ (50 nM) and monomeric Aβ_1–42_ (1 µM) were prepared in PBS, either combined or individually, in low-retention microcentrifuge tubes, and subjected to orbital shaking (750 rpm, 3 h at RT). Protein samples were then mixed with a non-denaturing buffer (Tris–HCl 63 mM, Glycerol 10%, bromophenol blue 0.01%), loaded onto 15% native gels (37:1 acrylamide:bisacrylamide ratio) and electrophoresed at constant 100 V in Tris–Glycine buffer. Proteins were subsequently transferred to PVDF membranes (0.45 µm pore size) at constant current (0.4 A) for 90 min, and the membranes were blocked in 5% milk. Primary antibody incubation was performed overnight at 4 °C using 1 µg/ml mouse anti-Aβ_1–16_ (6E10, Biolegend) and 400 ng/ml rabbit anti-ApoD (Proteintech), followed by incubation with HRP-conjugated secondary antibodies. Membranes were developed with ECL reagents (Millipore) and the signal recorded with a digital camera (VersaDoc; BioRad). The integrated optical density of the immunoreactive protein bands was measured with the FiJi program [35] in images taken within the camera linear range to avoid signal saturation.

### Ligand-binding assays by tryptophan fluorescence titration

Tryptophan fluorescence measurements were performed using a Shimadzu RF-5301PC spectrofluorometer equipped with a quartz cuvette (Hellma, 105.251-QS, 3 mm path length). All experiments were conducted at 25 ± 0.1ºC. Excitation was set at 295 nm to selectively excite tryptophan residues, and emission spectra were recorded from 300 to 400 nm with a slit width of 5 nm. Data acquisition and analysis were carried out using the RFPC software package (Shimadzu, Kyoto, Japan). ApoD^BCF^ was diluted to a final concentration of 0.5 µM in phosphate–EDTA buffered saline (10 mM phosphate, 150 mM NaCl, 1 mM EDTA, pH 7.0), ensuring that the intrinsic fluorescence signal remained within the optimal dynamic range. All samples were equilibrated in the dark for 3 min prior to measurement. Fluorescence spectra obtained in the presence of ligands were subtracted using control titrations with equivalent volumes of ligand-free carrier (1% NH_4_OH). The molecular autofluorescence of Aβ peptides was also subtracted. Corrected change in fluorescence intensity at 340 nm was plotted against ligand/protein ratio and fitted to the sum of two 3-parameter Hill equations, with *Kd* (apparent dissociation constant), *Fmax* (maximum fluorescence change), and *b* (degree of cooperativity) as the three parameters to be estimated by a least-squares method.

### Fluorescence microscopy

Aβ_1–42_ and ApoD^BCF^ 20 µl samples, either alone or in combination, were allowed to evolve at 4ºC for 10 days, deposited onto poly-L-lysine coated coverslips and incubated overnight in a humid chamber at 4ºC. Likewise, ApoD^Bact^ was incubated with Aβ_1–42_ for 24 h at 37ºC. The sedimented and attached proteins were fixed to the substrate with formaldehyde (4% in PBS) for 10 min at RT. After washing with PBS, samples were blocked with 10% goat serum in PBS for 1 h at RT. Primary antibodies incubation [1 μg/ml mouse anti-Aβ_1–16_ (6E10, Biolegend) or 20 μg/ml rabbit anti-ApoD (custom made by Abyntek Biopharma)] was performed for 2 h at RT. Alexa Fluor 488 or 594-conjugated IgG (Jackson Labs) were used as secondary antibodies. After washes in PBS, coverslips were mounted with Vectashield Vibrance^®^ (Vector Laboratories), and images were taken using an Eclipse 90i fluorescence microscope (Nikon) equipped with a DS-Ri1 digital CCD camera (Nikon). Images were acquired under the same conditions of illumination, diaphragm and condenser adjustments, exposure time, background correction and color levels, and analyzed using FiJi.

### Computational methods

Human ApoD atomic coordinates, including crystallographic water molecules, were obtained from the Protein Data Bank [36] (ID 2HZQ; 1.8 Å resolution; *R*_free_ 0.22). We used Modeller (v. 10.6) [37] to generate the native ApoD tertiary structure by homology modeling after mutated residues [38] were reverted to the wildtype primary sequence (file included as Supplementary File S2).

Aβ_1–42_ initial structures were generated by simulated annealing using Modeller. BLAST analysis [39] embedded in UCSF Chimera (1.19) [40] was used to find template PDB entries (7B3J, 7B3K, 7O1Q and 2BEG). Five AI-generated models of ApoD bound to Aβ_1–42_ were created with Protenix (https://protenix-server.com), and five using DeepMind AlphaFold-3 server (https://alphafoldserver.com/). These initial models served as tests for potential interactions and were followed by more reliable docking models obtained with HEX 3.1 [41], evaluating shape and electrostatics and performing energy minimization of selected structures using Jorgensen’s optimized potentials for liquid simulations [42].

Molecular Dynamics (MD) computations and analyses were performed with AMBER 24 package [43] under the 19SB forcefield [44] and using the SHAKE algorithm to constrain bonds involving hydrogen atoms [45]. Structures were solvated with OPC model water molecules [46], and counterions were added to neutralize total system charges. Secondary structures of Aβ_1–42_ were calculated with Kabsch and Sander’s algorithm [47] implemented in the CPPTRAJ module [48]. The protein moiety was energy-minimized, and the system was subjected to energy minimization with positional constraints for the protein main chain, followed by 400 ps MD under the isobaric-isothermal ensemble using Bussi’s thermostat [49] and a Monte Carlo barostat [50] to adjust box size and density, keeping main chain constraints. Then, we subjected the entire system to unconstrained energy minimization followed by 1 ns equilibration at 310 ºK and the subsequent production run (2.3 µs for Aβ_1–42_) under the NVT ensemble. For the Aβ_1–42_ peptide, a dual boost Gaussian accelerated MD (GAMD) [51] 10 µs trajectory was also computed after temperature equilibration, using a Langevin thermostat and friction coefficient of 1 ps^−1^. Trajectories of 500 ns were computed for the mixed models including 11 Aβ molecules with and without 1 molecule of ApoD. Analyses were performed with the CPPTRAJ module of AMBER24 and Origin (v2025; Originlab™). UCSF Chimera and ChimeraX enabled structural representations as well as further analyses. Errors are expressed as standard deviations.

### Statistical analysis

Statistical analyses were performed with GraphPad Prism (9.0), OriginPro and SigmaPlot (11.0) software. *p* < 0.05 was defined as a threshold for significant changes. *p*-values of statistical comparisons are reported in figure legends.

## Results

### Native human ApoD significantly delays Aβ_1–42_ aggregation

**Fig. 1:**
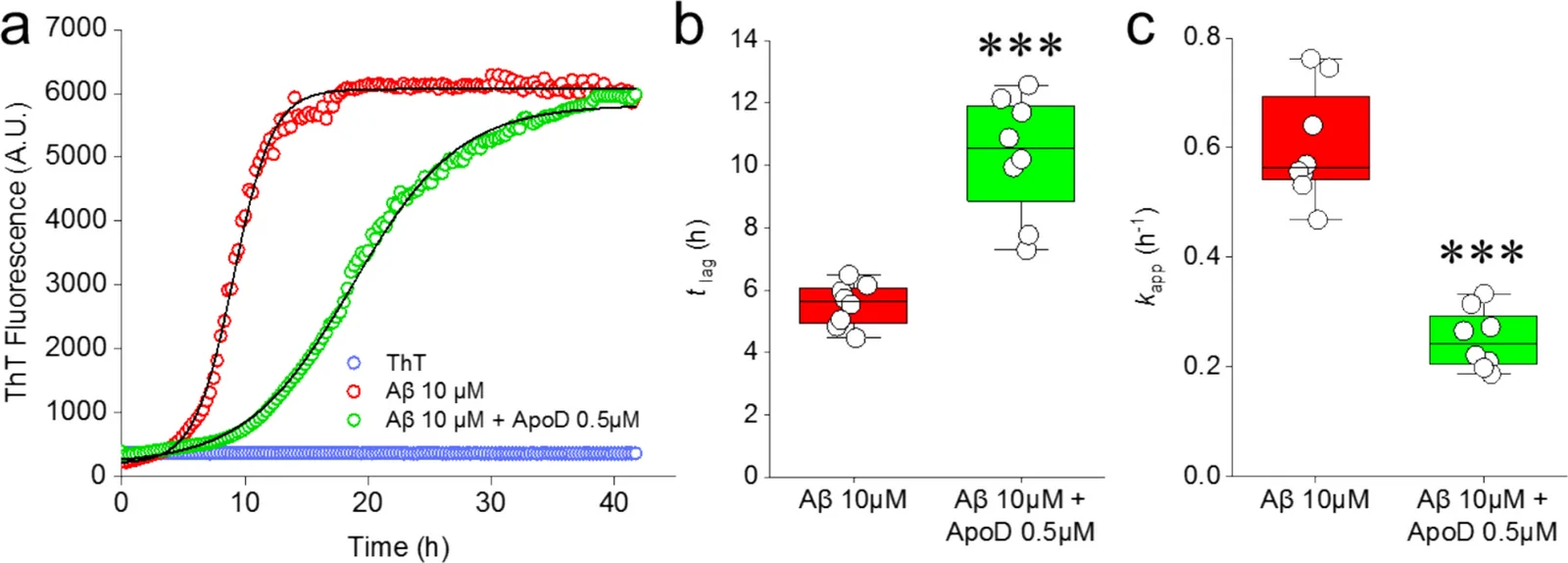
**Human ApoD significantly delays Aβ**_**1–42**_ **aggregation**. **a** Spontaneous aggregation of 10 µM Aβ_**1–42**_ in the absence (red dots) or presence of 0.5 µM ApoD.^BCF^ (green dots). ThT alone is shown in blue. Curves were fitted to a 4-parameter sigmoid function. **b-c** Aβ_1–42_ nucleation time (t_lag_) and elongation rate (k_app_) from n = 8 independent experiments. Box plots show median, quartiles 1 and 3 and minimum and maximum values excluding outliers. Asterisk points to significant differences (Student’s t-test; ***p < 0.001)

We performed in vitro ThT assays using purified human native ApoD (ApoD^BCF^) to test its ability to modify Aβ aggregation. We chose Aβ_1–42_ as it is the major component of amyloid plaques [52], with well characterized aggregate structure [53] and well-known propensity to aggregation, faster than the more abundant species Aβ_1–40_ [28, 54]. Following standard protocols [33, 34] we first established the conditions to monitor Aβ aggregation (Fig. S1). ThT was used at 20 µM [33], and we tested a range of 1–10 µM for Aβ peptides and 25–50 nM for ApoD^BCF^ (ratios ApoD/Aβ: 1/40–1/20). Control reactions included PBS, ThT, Aβ_1–42_ or each experimental protein alone (Fig. S1b), with no significant background fluorescence observed under these conditions. In situ static kinetic analyses at 37ºC were performed with 10 µM Aβ_1–42_ and 0.5 µM ApoD^BCF^ (ratio ApoD/Aβ 1/20; Fig. 1a). Aβ aggregation kinetics was analyzed by fitting fluorescence data to a four parameters sigmoidal function [34] and calculating the beginning of fibril elongation phase (*t*_lag_) and the apparent elongation rate (*k*_app_). The presence of ApoD^BCF^ altered the nucleation behavior of Aβ monomers, with an almost two-fold increase in the elongation lag phase (*t*_lag_) with respect to that of control aggregation (Fig. 1b), suggesting a significantly decreased probability of formation of ThT-positive oligomeric Aβ seeds required for fibril elongation. ApoD^BCF^ also reduced the elongation rate (*k*_app_) over three-fold (Fig. 1c), indicating a decreased velocity in Aβ fibril elongation. Final maximal ThT fluorescence is not altered by ApoD^BCF^, but is reached much later. Fluorescence of ThT alone did not change during the 40 h of incubation (Fig. 1a). These results indicate that ApoD, directly or indirectly, is able to influence both the early stages of Aβ aggregates formation and the elongation kinetics of fibrils. These are essential steps in the process, since the initial phases of Aβ dynamics are associated with the highest Aβ toxicity [8, 55].

**Fig. 2:**
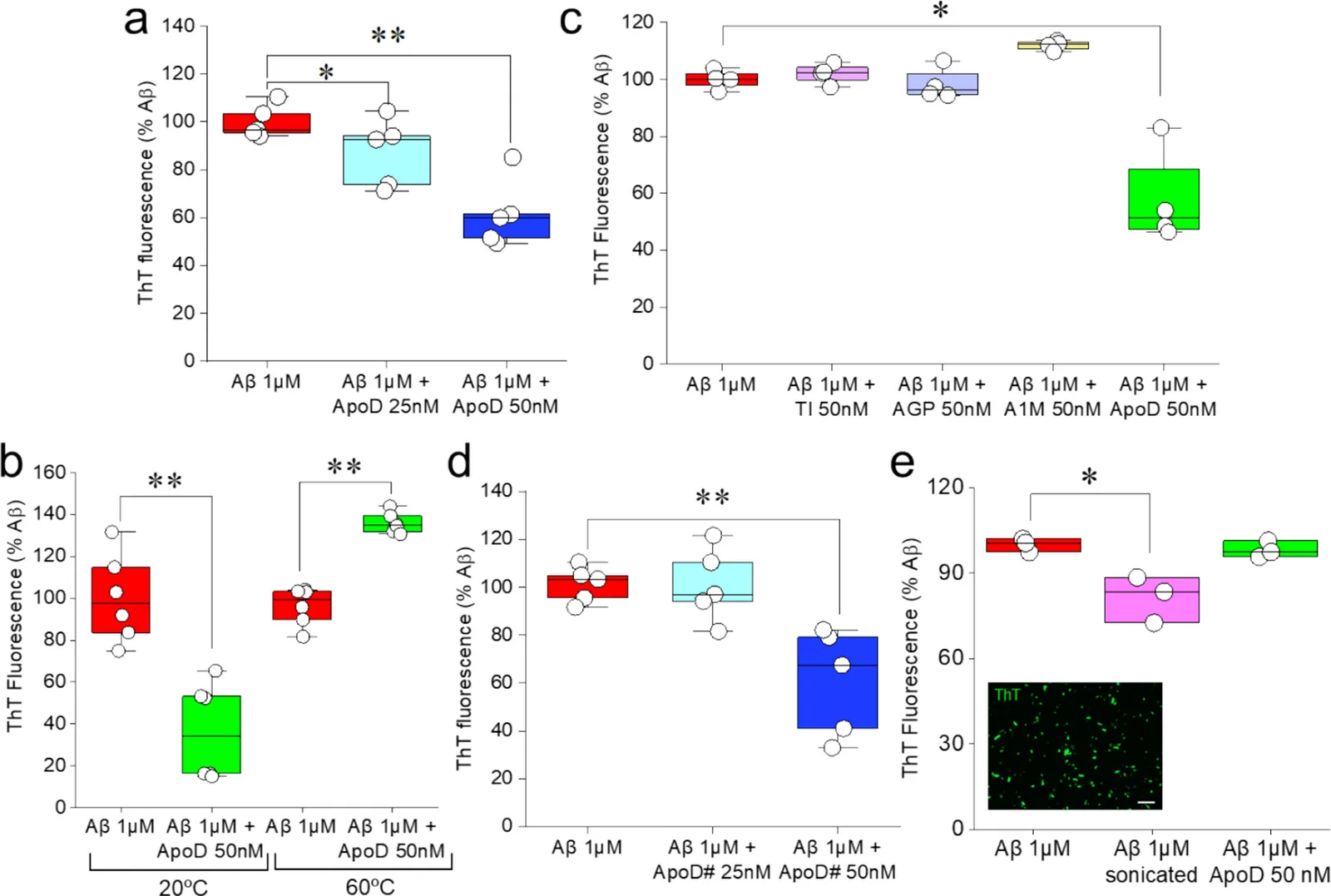
**Specificity and requirements for ApoD interference of Aβ**_**1–42**_ **aggregation or disaggregation processes**. **a** Aβ aggregation inhibition by native ApoD^BCF^ at 25 and 50 nM measured by ThT accelerated endpoint assay (3 h at RT with agitation). Data show a dose dependent effect. **b** Incubation temperature (20°C and 60 °C) does alter aggregation inhibition by ApoD^BCF^ (50 nM). Native folding of ApoD is therefore required for its inhibitory effect on Aβ aggregation. **c** Specificity test by comparison with different proteins: Trypsin Inhibitor (TI), Alpha-1-acid glycoprotein (AGP), Alpha−1 microglobulin (A1M), and ApoD^BCF^, all tested at 50 nM. Only ApoD interferes with Aβ aggregation. **d** ThT assay with recombinant ApoD^Bact^ (marked as ApoD#) at 25 and 50 nM. Glycosylation or oligomerization of ApoD are not required for its inhibitory effect on Aβ aggregation. **e** Disaggregation capacity assessed with pre-aggregated Aβ_1–42_ (1 μM monomer equivalent). A three min sonication pulse was used as positive control. No disaggregation activity is evidenced after 24 h incubation with 50 nM ApoD^BCF^. Inset shows ThT labeled Aβ aggregates at t = 0 (Scale bar 200 µm). Box plots show data distributions as in Fig. 1 from *n* = 3–6 independent experiments. Asterisks point to significant differences in relevant pairs (b) or with respect to control conditions (a, c, d, e) evaluated by ANOVA followed by Holm-Sidak pairwise comparisons. *p < 0.05; **p < 0.01

We used an accelerated aggregation endpoint protocol (3 h at RT) with 1 μM Aβ_1–42_ to test the specificity of the effect of ApoD. In these experiments, the ThT signal was normalized to the maximum fluorescence of the aggregated Aβ_1–42_ alone, and the effect on aggregation was calculated as the percent fluorescence reduction relative to control conditions. Adding ApoD^BCF^ at 25 and 50 nM (1/40 and 1/20 ApoD/Aβ ratios) resulted in a significant reduction of aggregates formation by 20 and 40%, respectively (Fig. 2a). These results demonstrate a concentration-dependent antiaggregating effect of ApoD. To assess whether the native three-dimensional conformation of ApoD is required for its effect on Aβ aggregation, ThT assays were conducted at 60 °C, a temperature known to denature other Lipocalins [56] without impairing Aβ_1–42_ aggregation [57] (Fig. S1d). Under these conditions, ApoD^BCF^ lost its antiaggregating effect (Fig. 2b), confirming that its native structure is necessary for functional interaction with Aβ_1–42_. The enhanced ThT fluorescence observed suggests a contribution of denatured ApoD to the formation of ThT-positive aggregates.

To further demonstrate the specificity of ApoD, we explored additional proteins with similar molecular weight or structural features. Soybean-derived trypsin inhibitor (TI), which has similar size but lacks the Lipocalin fold, and two human Lipocalins (Alfa-1-acid glycoprotein, AGP, and Alfa-1-microglobulin, A1M) were all tested at 50 nM. None of these proteins alter Aβ_1–42_ aggregation (Fig. 2c). To evaluate the role of post-translational modifications and oligomerization properties of ApoD on Aβ aggregation, we performed ThT assays with recombinant ApoD^Bact^ produced in *E. coli*, which lacks glycosylation and contains several modified residues [58] that prevent oligomerization, in contrast to ApoD^BCF^ that is able to tetramerize [59]. Although the anti-aggregating effect was less pronounced at 25 nM, 50 nM of ApoD^Bact^ is able to reduce Aβ_1–42_ aggregation by 40% (Fig. 2d, ApoD^Bact^ marked as ApoD#), suggesting that ApoD glycosylation and oligomerization are not required for its anti-aggregation function. 

We then evaluated whether ApoD could actively reverse Aβ_1–42_ aggregation by promoting disaggregation. ApoD^BCF^ was added to pre-formed Aβ_1–42_ aggregates, and ThT signal levels were monitored over a 24 h period. No reduction in ThT fluorescence was observed in the presence of ApoD^BCF^ (Fig. 2e). In contrast, a significant decrease was detected in the positive control condition, where Aβ_1–42_ aggregates were subjected to sonication in a water bath for 3 min before the final measure.

In conclusion, our findings support the hypothesis that the presence of ApoD during Aβ aggregation delays the onset of elongation and slows down the elongation process itself, without preventing the formation of aggregates that yield maximal ThT fluorescence. This suggests an interference at the early nucleation phase, decreasing the probability of oligomer formation, and that ApoD interference persists throughout the aggregation process. This effect does not require ApoD oligomerization or glycosylation, but ApoD must preserve its native conformation to exert this influence. Furthermore, we demonstrate that ApoD does not exhibit a disaggregation effect.

### ApoD colocalizes with Aβ_1–42_ plaques formed in vitro and modifies their structure.

**Fig. 3:**
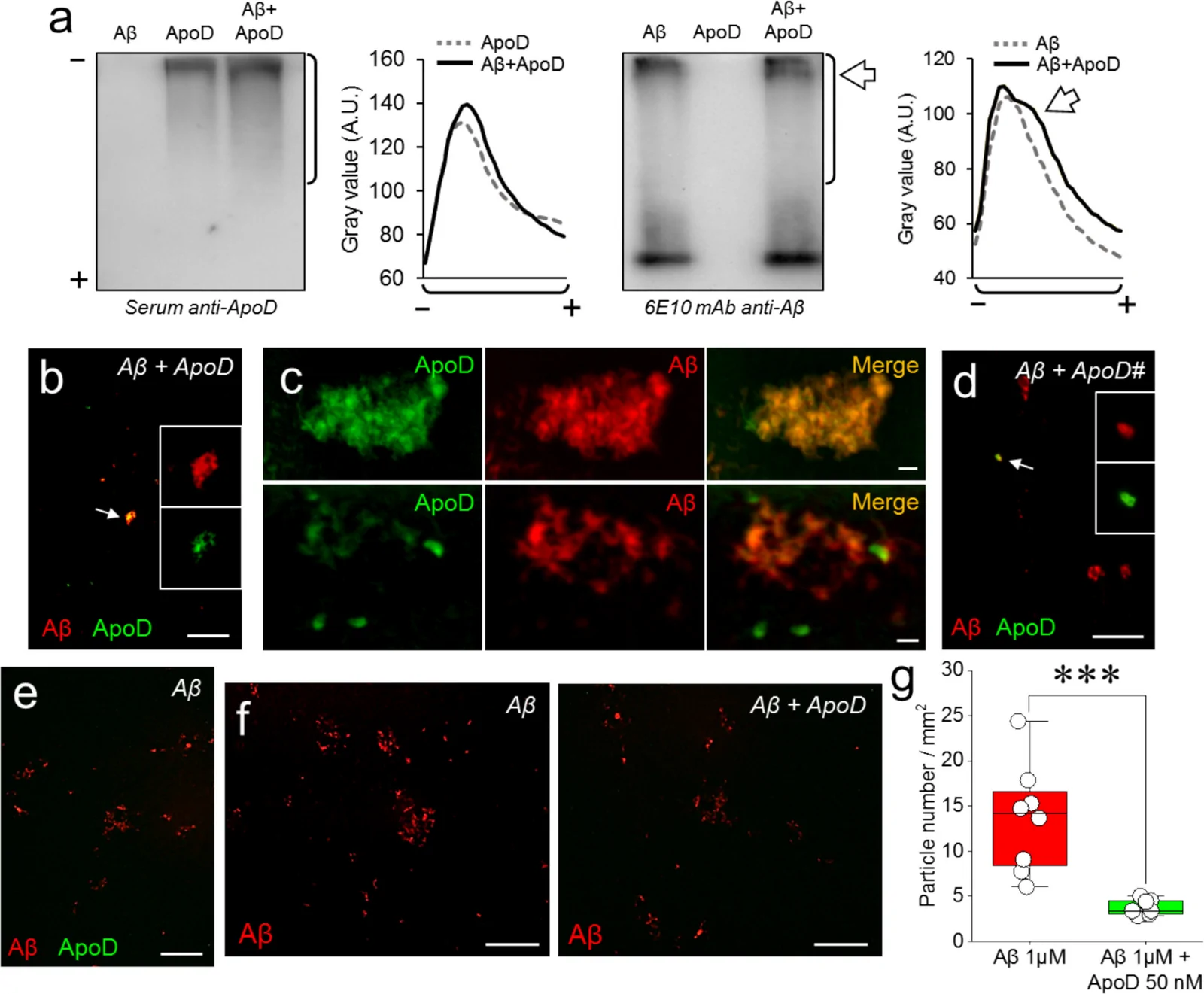
**ApoD colocalizes with in vitro Aβ plaques and alter their size profile**. **a** Analysis by native gel electrophoresis of ApoD and Aβ presence in aggregates formed after accelerated endpoint assays with 1 μM Aβ with or without 50 nM ApoD^BCF^. Electrophoretic mobility shows a switch to smaller/more compact aggregates positive for both antigens, particularly evident for Aβ (arrow). **b-d** Immunofluorescence microscopy images of Aβ_1–42_ aggregates (10 μM monomer equivalents) formed in vitro in the presence of 0.5 μM ApoD^BCF^ or ApoD^Bact^ (marked as ApoD#). **e** Negative control for ApoD antibody on in vitro plaques. Antibodies against ApoD give no signal in samples with only Aβ. **f-g** The number of Aβ aggregate particles sedimented on the coverslips decrease in the presence of ApoD.^BCF^. Significant difference determined by Student’s t-test, ***p < 0.001. Scale bars: 20 μm (b, d, e, f); 2 μm (c)

Having established that ApoD is able to reduce Aβ aggregation rate, we hypothesized that this effect must be based on a direct interaction between the two proteins. Aβ_1–42_ aggregates induced by orbital agitation (3 h) show a change in electrophoretic mobility when exposed to ApoD^BCF^ that is evidenced in immunoblots after native polyacrylamide gel electrophoresis (Fig. 3a). A new high molecular weight band appears, which indicates a change in either mass, charge or compactness of the Aβ_1–42_ aggregates formed.

Following Trp fluorescence experiments (see below), the solutions of Aβ_1–42_ alone or incubated with ApoD^BCF^ for 10 days were dropped onto poly-L-lysine coated glass coverslips, and aggregates were allowed to settle down before formaldehyde fixation to the coating matrix and subsequent washing of free moving proteins. The immunodetection of ApoD and Aβ_1–42_ revealed a clear colocalization of both proteins in the deposited aggregates (Fig. 3b and c), ranging from partial (Fig. 3c lower panels) to almost full colocalization (Fig. 3c upper panels). ApoD^Bact^ also co-localizes with Aβ_1–42_ aggregates formed in vitro for 24 h at 37ºC (Fig. 3d, marked as ApoD#). The antibody against ApoD showed no specific signal on samples with only Aβ (Fig. 3e). Moreover, the quantification of the immunodetected Aβ_1–42_ aggregates fixed to the coverslip surface revealed that their number significantly decreases when ApoD^BCF^ is present during the Aβ aggregation process (Fig. 3g and h). Since ApoD does not affect the maximal ThT fluorescence (Fig. 1a) our results indicate that the formation of large aggregates is reduced in the presence of ApoD, while small ones persist but they would sediment poorly on the coverslips. This is also coherent with the profile of Aβ detected in the native electrophoresis (Fig. 3a). Together these results demonstrate that both ApoD^BCF^ and ApoD^Bact^ can stably interact with Aβ_1–42_ in plaques formed in vitro and support a direct interaction of ApoD and Aβ_1–42_ in solution, contributing to slow down the initiation and growth of peptide aggregates that otherwise would enlarge and tend to sediment on the matrix.

### ApoD tryptophan fluorescence assays demonstrate complex direct ApoD-Aβ interactions, compatible with initial AI-based rigid docking models.

**Fig. 4:**
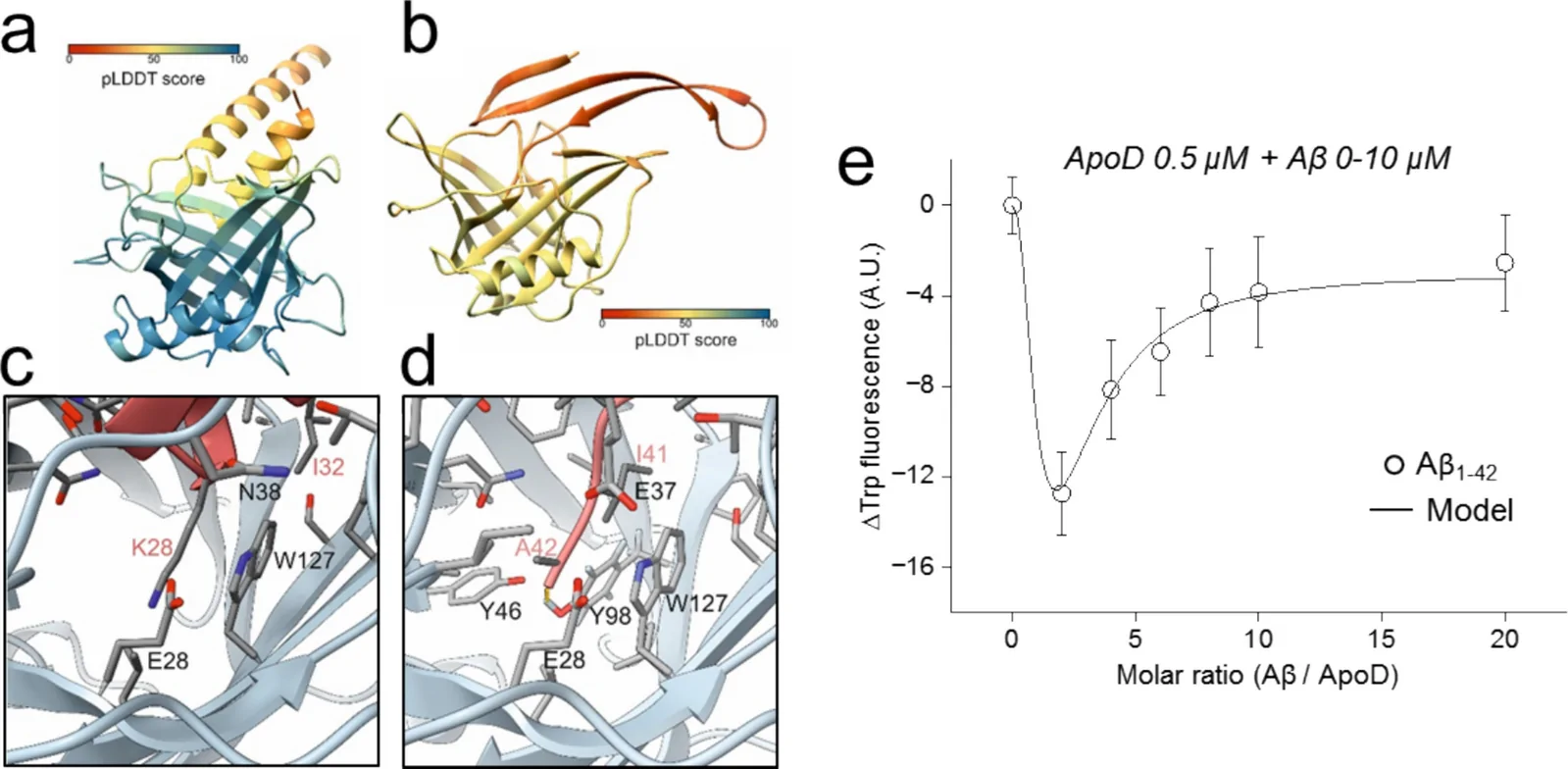
**Aβ**_**1–42**_ **interacts with ApoD in a complex manner, unexpected from simple docking models**. a-d AI-driven models of the ApoD-Aβ1–42 complex. Cartoon diagrams of the two partners. a AlphaFold3 first model (out of five best docking solutions). b Protenix first model (out of five docking solutions). Ribbons are colored according to the Local Distance Differences Tool prediction (pLDDT) scores in each program. c-d Close up views of the portion of Aβ interacting with the ligand-binding pocket of ApoD in each model. c In the AlphaFold3 model Aβ Asn27 clearly collides with ApoD Ile70 and Aβ Lys28 holds Van der Waals and hydrophobic interactions with ApoD Trp127. d In the Protenix model, a hydrogen bond forms between Aβ C-terminus and ApoD Tyr98 Hζ (hydroxyl), while Trp127 is buried and two nearby carboxylates (Glu37 and Glu28) may exert repulsive forces. e Tryptophan fluorescence titration (λ_Ex_ 295 nm; λ_Em_ 340 nm) performed with 0.5 µM ApoDBCF and Aβ 1–10 µM. Average ± SD is plotted (n = 3) against Aβ/ApoD molar ratio. Experimental data are fitted to the sum of two independent 3-parameters Hill equations (see Fig. S4 for details). Changes in Trp127 fluorescence, located in the pocket, cannot by itself explain the behavior of ApoD binding to Aβ monomers

All previous ligands described for ApoD were small hydrophobic molecules [reviewed by 13] bound to the pocket shared by all Lipocalins. As a first approximation to understand the potential interaction between ApoD and Aβ_1–42_, we studied molecular models generated by AlphaFold3 and Protenix, two different protein structure prediction AI-based applications, being aware that the Protenix models yielded low per-residue confidence scores, measured by the predicted per-residue Local Distance Difference Test-Cα [60]. Both systems predict Aβ-ApoD complexes based on an interaction of Aβ with the hydrophobic pocket of ApoD, but they differ in the Aβ_1–42_ conformation: AlphaFold3 predicts an all-α conformation (Fig. 4a), while Protenix predicts an all-β conformation (Fig. 4b). In both models Aβ_1–42_ fills the hydrophobic pocket of ApoD and buries Trp127 indole group, though in a different manner. Aβ_1–42_ bends itself leading to two α-helices in the AlphaFold3 model, where Aβ Asn27 clearly clashes with Ile70 in ApoD and Aβ Lys28 holds Van der Waals and hydrophobic interactions with ApoD Trp127 (Fig. 4a and b; Fig. S3a). Therefore, the AlphaFold3 model suggests an interaction of ApoD with the central moiety of Aβ peptide, known to be especially important for stabilization of intermolecular interactions within Aβ fibrils [61]. In contrast, in the Protenix model the C-terminal end of Aβ_1–42_ is the one entering the binding pocket and forming a hydrogen bond with Trp127 at the ApoD pocket bottom (Fig. 4c and d; Fig. S3b).

The predicted interaction with Trp127, and the lack of Trp residues in Aβ_1–42_, prompted us to experimentally test Aβ- ApoD^BCF^ binding by Trp fluorescence titration analysis (λ_Ex_ 295 nm, λ_Em_ 340 nm), a method already used to study ligand binding in many Lipocalins [62] and ApoD [32]. The titration curve exploring several ApoD/Aβ ratios (1/2–1/20; 0.5 μM ApoD, 1–10 μM Aβ) reveals a unique and complex mode of interaction (Fig. 4e). This result suggests more than one binding site, with at least one with high affinity that decreases Trp fluorescence, and at least a second site with low affinity increasing fluorescence. The experimental data are well fitted by the sum of two 3-parameter Hill equations with estimated Kd of 0.9 and 3 µM respectively, and a similar degree of cooperativity (Fig. 4e; Fig. S4a). Thus, Trp127 located in the pocket cannot by itself explain the behavior of ApoD binding to Aβ_1–42_ monomers, and other Trp residues must be involved. This result led us to perform a more exhaustive analysis of the predicted molecular dynamics of Aβ species alone and in the presence of ApoD.

### ApoD has multiple binding sites with differential affinities for N- and C-terminal ends of Aβ, as evidenced by tryptophan fluorescence after predictions by dynamic modeling of monomeric Aβ

**Fig. 5:**
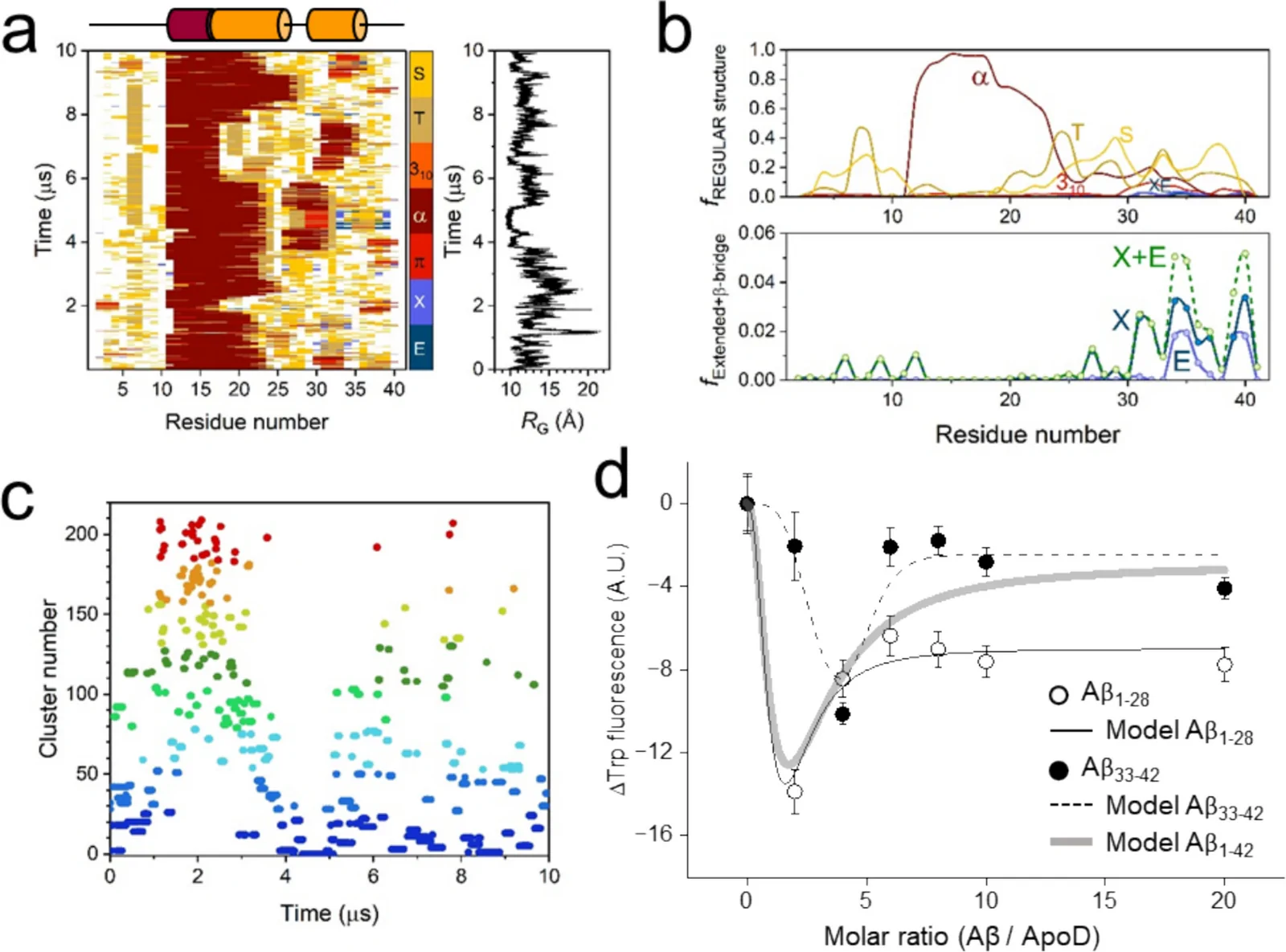
**Gaussian-accelerated Molecular Dynamics of Aβ**_**1–42**_ **leads to test ApoD binding to N- and C-terminal fragments of Aβ**_**1–42**_. **a** Secondary structure of Aβ_1–42_ timeline along the 10 µs GA-MD trajectory. The RMSD of the mainchain with respect to the initial energy minimized structure is 9.66 ± 1.65 Å. One or two major α-helices visibly predominate as most frequent structures. Right plot shows the evolution of the radius of gyration (R_G_) along the trajectory displaying the strong variations of peptide compactness. **b** Per-residue frequencies of the various secondary structure elements calculated along the trajectory. General view in upper panel. Lower panel shows an enlarged view of extended and β secondary structures mostly concentrated at the C-terminal portion of the peptide. **c** Cluster analysis of the 10 µs GA-MD trajectory. **d** Tryptophan fluorescence titration (λ_Ex_ 295 nm, λ_Em_ 340 nm) performed with 0.5 µM ApoD^BCF^ and Aβ_1–28_ or Aβ_33–42_ fragments (1–10 µM). Average ± SD is plotted (*n* = 3) against Aβ/ApoD^BCF^ molar ratio. Experimental data are fitted to the sum of two independent 3-parameters Hill equations (see Fig. S4 for details). Both fragments yield titration curves with two components, indicating their ability to bind to multiple sites of ApoD and influencing more than one Trp residue. Model of binding to Aβ_1–42_ (in gray) is shown for comparison. The high affinity component that quenches ApoD Trp fluorescence is similar in Aβ_1–28_ and Aβ_1–42_ titration curves

We first modeled the behavior of Aβ_1–42_ peptides starting from homology models of the membrane-bound full APP protein, which would be cleaved to obtain the Aβ_1–42_ fragment. This yielded mostly a α-helix based structure. Two α-helical regions were stable in classical MD simulations (cylinders in Fig. S5a), with amino acids 23–28 forming a flexible loop between them. The second helix was unstable during GAMD computations (light brown cylinders in Fig. 5a) where conformational changes are favored by decreasing rotational transition state energies. The first helix remained stable along the times explored (MD: 2.3 µs; GAMD: 10 µs). Since Aβ_1–42_ results from cleavage of APP in its transmembrane α-helical domain, these models mimic putative conformations acquired by the peptide soon after it is shed from cell membranes. Simulations show an overall highly dynamic conformation exchange. The radius of gyration (*R*_G_), which measures the peptide compactness, fluctuated more than 30% during the analyzed trajectories (Fig. 5a and S5a). Root mean square deviation (RMSD) values with respect to the initial energy-minimized structure reached a highly fluctuating plateau (9.66 ± 1.65 Å for main chain atoms after the first 89 ns of the 10 µs GAMD trajectory). Furthermore, hierarchical clustering yielded more than forty distinct groups with the classical MD simulations. The most stable groups were sampled during several hundreds of ns (blue dots in Fig. S5c), and three of them appear only twice in very separate periods of the trajectory (red dots in Fig. S5c). A total of 209 clusters were classified in the GAMD trajectory (Fig. 5b). The fraction of extended (β) and β-bridge (stabilized by hydrogen bonds) conformations that were sampled during the computations (blue and purple in Fig. 5a, b and S5a, b) was very small and mostly concentrated at the C-terminal part of the peptide (0.020 ± 0.018 on average for the last 16 residues) (Fig. 5b and S5b, lower plots). As expected, no switch to mostly-β structures happened within the times explored.

The dynamic modeling of Aβ_1–42_ indicated that its C‑terminal region displays high flexibility and may play a relevant role in the interaction of Aβ with ApoD. The model generated by Protenix, though with low per-residue confidence scores, also suggests an interaction of the C-terminal end of Aβ_1–42_ with ApoD. These observations prompted us to experimentally assess whether one of the multiple Aβ-ApoD interactions revealed by Trp fluorescence titration is specifically mediated by the C-terminal region of the peptide. To address this question, we performed Trp fluorescence titration using the isolated C‑terminal fragment Aβ_33–42_, and compared its behavior with Aβ_1–28_, a truncated peptide lacking the C‑terminal region (Fig. 5d; Fig. S4b-c). To our surprise both fragments also yield distinct titration curves with two components, retaining their ability to bind to multiple sites of ApoD^BCF^. Trp fluorescence using both peptides suggests high-affinity and low-affinity binding sites with opposing effects, but with different estimated Kd and cooperativity. Interestingly, the estimated Kd of the Aβ_1–28_ high affinity component (0.9 µM) equals that of the full Aβ peptide (model displayed in gray for comparison), strongly suggesting that they share at least one of the binding sites in ApoD. In contrast, the Aβ_1–42_ curve is more similar to that of Aβ_33–42_ in the upper concentration range. Together these results suggest that different segments of the Aβ peptide alter the fluorescence of different Trp residues in ApoD, with at least one high affinity site of interaction shared by the full peptide and Aβ_1–28_, and a different one with less affinity shared by the full peptide and Aβ_33–42_.

Since the central moiety of Aβ is known to interact with other lipocalins [30, 63], and it is common to Aβ_1–42_ and Aβ_1–28_, we hypothesize that Aβ_1–42_-ApoD interaction should be modified if we use a mutated version of Aβ_1–42_ (Aβ_1–42_-K28A) for modeling (Fig. S6). Curiously, the predicted interactions of the mutant Aβ favor binding through its C-terminal end.

### Dynamics modeling with ApoD/Aβ at different ratios predicts that ApoD decreases the probability of β structures at the C-terminal end of Aβ_1–42_

Since both the aggregation and binding assays have been performed with Aβ concentrations always in excess to ApoD, we modeled putative complexes at different ratios (1/1 and 1/11 ApoD/Aβ), mirroring the concentration ranges used in ThT and Trp fluorescence titration experiments.

**Fig. 6:**
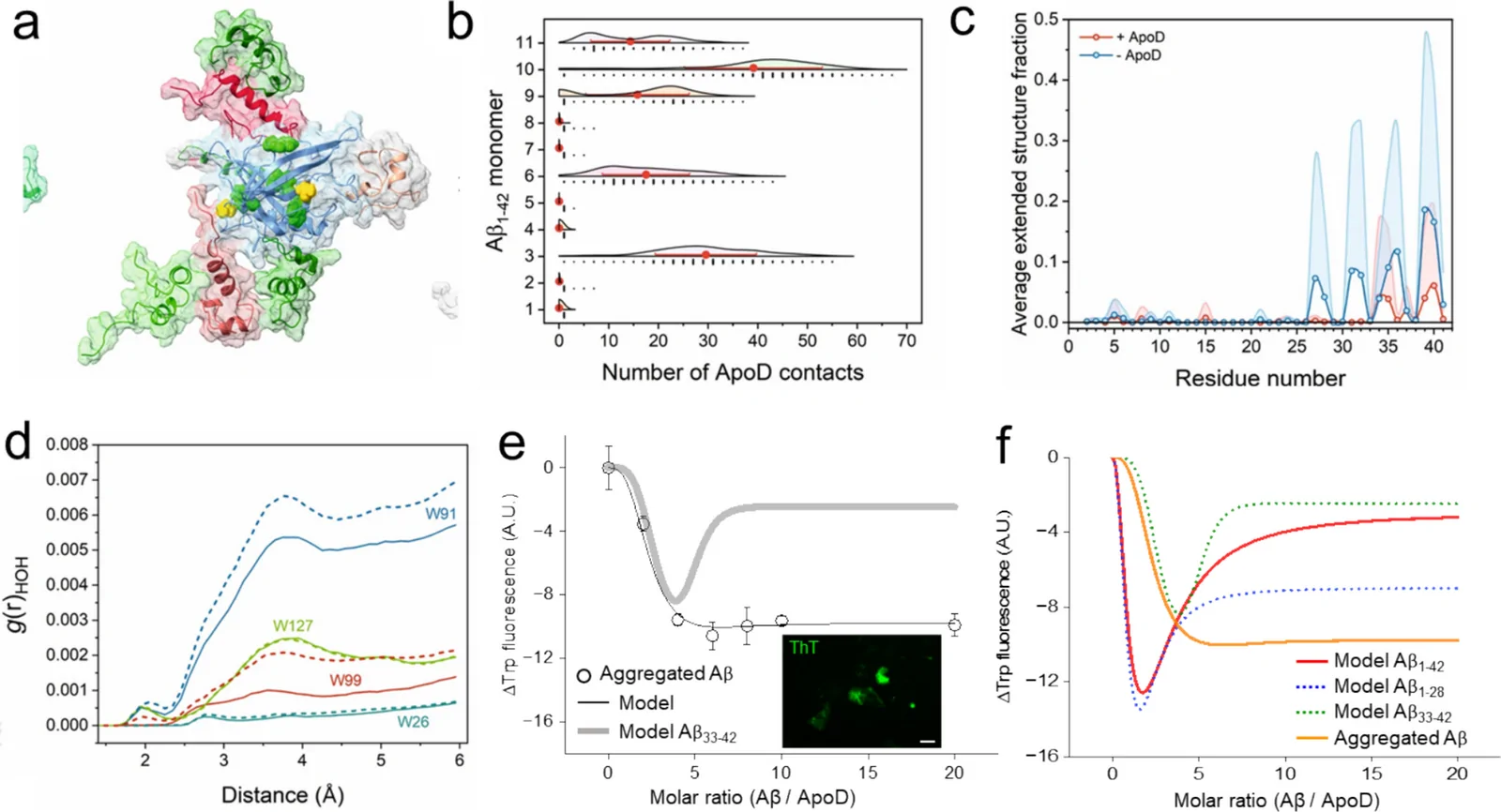
**ApoD decreases the probability of Aβ peptide transition to β conformations and modifies its interaction to a simpler form once the peptideis aggregated**.** a** Partial view of the energy-minimized, closest to the average, structure snapshot of the computation including ApoD and eleven Aβ molecules in a 3.65 × 10^–3^ μm.^3^ modeling box. ApoD is shown in blue, its Trp residues in green, and glycosylated Asn45 and Asn78 in yellow. Distinct Aβ_1–42_ monomers are shown in different colors. Four monomers are directly bound to ApoD at different positions: two are forming ternary contacts to bound Aβ_1–42_ monomers; five are free at the rim, while most peptides are out of the ApoD region. A set of five selected timelines showing the diversity of behavior of Aβ monomers, with and without ApoD in the box, are shown in Fig. S9. **b** Per-monomer distribution of the number of contacts with ApoD along the trajectory. A cut-off of 6 Å between α-carbons was selected to define the existence of a contact. Red circles and red lines (whiskers) represent mean and standard deviation values for each distribution. Ternary complexes and complexes between Aβ_1–42_ monomers are not considered in this analysis. **c** Per-residue fractions of extended/β-bridge structure computed along the trajectory for the eleven Aβ_1–42_ monomers along the MD computations. Line-points represent the average for the 11 structures, and shadowed lines represent standard deviations. The results without ApoD (blue plot) and with ApoD (red plot) are statistically different (U-test; p = 0.027). **d** Radial distribution functions of water molecules (g(r)_HOH_) around ApoD Trp residues, as computed from MD trajectories. Slash and solid lines correspond to ApoD in the absence or presence of Aβ_1–42_ respectively. **e** Tryptophan fluorescence titration (λ_Ex_ 295 nm, λ_Em_ 340 nm) performed with 0.5 µM ApoD and pre-aggregated Aβ_1–42_ (1–10 µM monomer equivalents). Average ± SD is plotted (*n* = 3) against Aβ/ApoD molar ratio. Experimental data can be modeled by a predominant component quenching Trp fluorescence, with a small component increasing fluorescence (see Fig. S4 for details). Model of binding to Aβ_33–42_ is shown in gray for comparison. The component that quenches ApoD Trp fluorescence upon Aβ_33–42_ binding is similar to that obtained with pre-aggregated Aβ_1–42_

The AI-generated models could represent final states for 1/1 complexes, but we were interested in testing interactions for the initial Aβ states. Rigid docking using representatives from the best-populated clusters from the Aβ monomers MD simulation (Fig. S5c) resulted in a broad variety of solutions (Fig. S7a), most of which interact with ApoD at sites different from the ligand binding pocket. We then performed MD simulations at 1/11 ratio, with Aβ_1–42_ molecules representing conformations of the first eleven GAMD clusters (Fig. 5c). They were introduced together, with or without a single ApoD molecule, in a 3.65 × 10^3^ nm^3^ modeling box. Seven of the eleven Aβ_1–42_ monomers directly contacted ApoD at different times of the trajectory, some of them simultaneously. Higher order complexes were also detectable (Fig. 6a). The Aβ monomers interacting with ApoD along the trajectory display varying numbers of contacts (Fig. 6b). One of the eleven Aβ molecules displays a stable position bound to the rim of ApoD binding pocket (cyan in Fig. S7b) in the MD trajectory explored, despite being highly dynamic (see supplementary Video S8).

Interestingly, even with our modeling time limitations, the presence of ApoD correlates with a 2.5-fold decrease in β structures content (0.055 ± 0.059) at the C-terminal end of Aβ_1–42_ (Fig. 6c and S9), suggesting that ApoD could decrease the probability of a transition to β conformation in the Aβ peptide, a result coherent with the apparent increase in *t*_lag_ obtained in the aggregation assays.

### Upon aggregation, interactions of Aβ with ApoD change from multiple to a single binding site involving the C-terminal end of Aβ

Analyzing the behavior of the four Trp residues present in ApoD, a decrease in solvent accessibility of ApoD Trp91 and Trp99 is predicted, providing an explanation for the experimental increase in fluorescence of ApoD upon exposure to increasing concentrations of Aβ_1–42_, Aβ_1–28_ and Aβ_33–42_. In contrast, no change in the distribution of water molecules around Trp26 or of the binding pocket located Trp127 is evidenced in the timeframe modeled (Fig. 6d). The location of the four Trp residues of ApoD can be seen in Fig. 6a (green spheres).

Since ApoD interferes Aβ aggregation both at early stages and along the process, until a new equilibrium with maximal aggregation is reached, we decided to test ApoD binding to aggregated Aβ_1–42_ peptides. Trp fluorescence titration assays of ApoD exposed to pre-aggregated Aβ (Fig. 6f) demonstrate that once the switch to aggregation-prone β conformations of Aβ_1–42_ has taken place, ApoD-Aβ interaction is simplified to a form where a decrease in Trp fluorescence is the predominant effect, with only some residual increase at high Aβ concentrations (Fig. S4d). The model obtained with Aβ_33–42_ is shown in gray for comparison, revealing that the ApoD Trp that decreases its fluorescence upon binding to aggregated Aβ must interact with the C-terminal region of Aβ with a similar estimated Kd (2.8 and 2.4 µM respectively). Thus, ApoD-Aβ interactions change from a multiple site mode of binding, altering the fluorescence of Trp residues in opposing directions, to a single form of binding involving the Aβ C-terminus, which is predicted to be available as extended structure in the all-β conformation acquired by the peptide through its aggregation process. The modeling and experimental binding data together points to Trp127, within the classical Lipocalin binding pocket, as the candidate for this single site binding mode.

## Discussion

A detailed understanding of Aβ aggregation is becoming increasingly important [64], particularly since the initial hypotheses of amyloid plaques being the harmful agents in an AD brain was challenged and the intermediate oligomeric forms of Aβ were discovered to be responsible for most damaging effects reviewed by [65]. Many pharmacological agents have been described [66–69], and fourteen proteins are currently annotated with the ontology term “negative regulators of amyloid β formation” (GO:1905907) [70]. Additionally, up to eighteen other proteins with experimental evidence of Aβ anti-aggregation activity have been reported [28, 71–73]. Ten of them are located extracellularly and, like ApoD, three show lipid-binding capability or are associated to membranes or lipid structures. Proteins in this category share biophysical properties suitable for binding both Aβ peptides and lipidic structures. They can therefore interfere with Aβ toxic effects in varied ways: modulating APP processing in membranes, influencing oligomerization and aggregation of the hydrophobic peptide once shed from APP, and transporting it out of the CNS or into cellular organelles for degradation. ApoE, ApoA-I and ApoJ are well established members of this functional category [reviewed by [31]. The present study provides compelling evidence that ApoD, one of the few genes consistently overexpressed upon physiological aging in mammals [13], belongs to this group of proteins exerting a negative modulation of the Aβ aggregation process. These findings must trigger interesting lines of research exploring how ApoD-Aβ interactions are modulated by the presence of the diverse lipids and lipidic structures relevant in the aging brain.

Our findings demonstrate that ApoD significantly delays the nucleation and elongation phases of Aβ fibrillogenesis and that it is able to directly interact with Aβ in solution in its initial monomeric state, as demonstrated by Trp fluorescence experiments. These findings suggest that a major influence of ApoD occurs during the early stages of Aβ aggregation, slowing this process approximately twofold. ApoD ability to chaperone Aβ in these early phases would prevent Aβ damaging effects on cells, a prediction coherent with the neuroprotective effects of ApoD in vivo, where brain aging is accelerated in mice lacking ApoD [74].

The inhibition of aggregate formation by ApoD is concentration-dependent, and its effect is retained by bacterial recombinant ApoD. Also, both forms of ApoD (ApoD^BCF^ and ApoD^Bact^) can interact with Aβ and co-localize with aggregates formed in vitro. The recombinant ApoD^Bact^ lacks glycosylation and oligomerization ability [58], indicating that these post-translational modifications are not essential for its effects on Aβ dynamics. Our results predict that most forms of native ApoD, whose glycosylation and oligomerization varies depending on physiological context and sites of expression [59, 75, 76], would retain Aβ chaperone and anti-aggregation roles. Furthermore, thermal denaturation abolished ApoD activity, underscoring the requirement for a native conformation to mediate this effect.

In contrast, ApoD neither exhibits disaggregating activity on preformed Aβ fibrils nor prevents the formation of distinct aggregates. This finding is coherent with the specific interaction of pre-aggregated Aβ with ApoD demonstrated by Trp fluorescence titration, and with the co-localization of ApoD with in vitro formed aggregated structures. However, the presence of ApoD does alter the size distribution of aggregates, reducing the number of large aggregates. Therefore, ApoD is affecting not only the initial probability of oligomerization and fibril elongation, but also the final size and structure acquired by the amyloid plaques. These results explain the observed distribution of ApoD in compact plaques of human brain samples [26, 27, 77], and agree with ApoD-KO mice on an APP/PS1 background exhibiting significantly larger plaques than controls [21]. Future studies using transmission electron microscopy (TEM) directly monitoring amyloid fibril formation in the presence of ApoD should help to determine whether ApoD also alters the ultrastructural organization and morphology of the resulting fibrils, as it has been previously described for other lipocalins [30].

Our strategy combining modeling and experimental test of ApoD-Aβ interactions yields an unexpected picture about how ApoD modulates Aβ aggregation. It must be emphasized that we have used native human ApoD and synthetic Aβ peptides without modifications, thus allowing any type of interaction to take place in the binding or aggregation assays.

Initial docking models, albeit with low-confidence scores, propose interactions of Aβ within the ApoD hydrophobic pocket that predicts changes in ApoD Trp127 fluorescence, as described for small hydrophobic ligands of ApoD [32]. However, we obtained titration curves markedly different from the classical one-site saturation curves derived from a single binding site. Our fluorescence titration models predict at least two binding sites, well fitted to 3-parameters Hill equations. Two different Trp residues are therefore required to explain the observed behavior: a high-affinity site causing fluorescence quenching at low Aβ concentrations, and another site with less affinity that increases Trp fluorescence. Each binding site is best modeled if differing degrees of cooperativity are introduced in the fitting parameters, suggesting that interactions of one part of the peptide might promote subsequent interactions with nearby ApoD regions.

These interesting forms of interaction led us to a deeper analysis of Aβ-ApoD docking by classical and Gaussian accelerated MD simulations. We first modeled the potential conformations that might be acquired by Aβ once is released of cell membranes after cleavage from APP. Our predictions indicate that Aβ retains a substantial α-helical content, with one or two α stretches separated by loops of different sizes and a C-terminal region (Aβ_33–42_) exhibiting greater flexibility and propensity for extended conformations. Aβ does not reach sufficient transition energy [78] to adopt β-sheet conformation within the limited simulation timescale, agreeing with previous reports [79].

Docking simulations at ApoD/Aβ ratios of 1/1 and MD simulations at 1/11 demonstrated multiple potential interaction sites and the possibility of simultaneous binding of several peptides, including sites different from the classical ligand-binding pocket, and thus coherent with the complex Trp fluorescence titration curves obtained. Trp fluorescence titration experiments using truncated peptides (Aβ_33–42_ and Aβ_1–28_) also yield minimal models with at least two binding sites.

The behavior of Trp-fluorescence upon Aβ_1–28_ and Aβ_1–42_ binding to ApoD, and the analysis of the dynamic modeling trajectories performed, suggest that Aβ is able to bind to the ApoD pocket through the flexible portion between the two Aβ α-helices evidenced in our GAMD simulation (colored cylinders in Fig. 5a). This portion includes residues 18–28, common to both peptides. This scenario would be comparable to the initial docking model generated by AlphaFold3. Interestingly, a similar mode of binding has been captured in the crystal structures of Aβ_1–40_ complexed with two Anticalins, H1GA and US7 [63], which are engineered Lipocalins generated by artificial selection starting from variations of Lipocalin 2 (LCN2) [80]. Since the Aβ peptide crystalized with Anticalins was biotinylated at its C-terminus, other binding modes might have been disfavored. However, the crystal structures clearly demonstrate the feasibility of this particular mode of binding. Notably, the weakness of the electron density for the rest of Aβ residues in the crystals with Anticalins strongly supports that Aβ_1–40_ remains highly dynamic upon binding, similar to our modeled trajectories where Aβ_1–42_ interacts transiently but repeatedly with ApoD while keeping its dynamic conformational exchange, even forming ternary complexes. Titration of ApoD with both Aβ_1–42_ and Aβ_1–28_ predicts a site of similar low Kd (< 1 µM) and cooperativity (> 2) that reduces Trp fluorescence upon binding, indicating that the predominantly α initial conformations of Aβ_1–42_ monomers probably interact with the binding pocket of ApoD in a way similar to Anticalins. However, both peptides must have contacts with additional ApoD Trp residues. Water radial distribution measured during MD computations indicated solvation changes for ApoD Trp91 and Trp99 that could explain the experimental fluorescence increases upon exposure to Aβ_1–42_ and Aβ_1–28_. In fact, Trp99 is one of the mutated residues in ApoD^Bact^ [58], thus offering a plausible explanation for the slightly lower anti-aggregation effect of this recombinant form of ApoD.

In contrast, binding to the C-terminal fragment (Aβ_33–42_) displayed distinct binding kinetics, requiring higher peptide concentrations to alter fluorescence in both directions. None or minor changes in the solvent structure around Trp127 or Trp26 were observed within the simulation timeframe. Interestingly, titration of ApoD with pre-aggregated Aβ demonstrates that once the switch to aggregation-prone β conformations has taken place ApoD–Aβ interaction simplifies to a form where fluorescence quenching predominates. Comparisons of titration curves and models indicate that aggregated Aβ must bind primarily through its C-terminus (as predicted by the Protenix model or when Lys28 is mutated in Aβ_1–42_), with a high Kd (> 2 µM) similar to the first component of the Aβ_33–42_ titration curve. Thus, along the aggregation process, interactions of ApoD with Aβ shift from a multi-contact binding mode that includes binding to the central moiety of Aβ, to a single-site interaction involving its C-terminal end. In both types of interaction, the classical ligand-binding pocket of ApoD is involved, though in a different manner.

Our MD analysis simulating excess of Aβ with respect to ApoD, has also allowed us to extract estimates of probability of appearance of a particular conformation or interaction. Despite the limited simulation time, the GAMD predicts that ApoD partially mitigates the transition toward β-enriched structures. This behavior supports that ApoD prolongs the time Aβ remains in conformations less favorable for fibril formation, providing a coherent explanation of the ApoD-caused delay in Aβ aggregation experiments.

The specificity of ApoD-Aβ interaction was supported by the absence of anti-aggregation activity in unrelated proteins of similar molecular weight (Trypsin inhibitor) or in evolutionary and structurally related Lipocalins. A1M is mainly expressed by hepatocytes and has not been associated with Aβ [81]. AGP is a biomarker of cerebral amyloid angiopathy [82], but its role in Aβ aggregation had not been explored previously. Here we demonstrate that both Lipocalins do not show anti-aggregation function in vitro in the condition tested. In contrast, anti-aggregation effects have been demonstrated for another Lipocalin expressed by brain cells, L-PGDS [28] and two Anticalins derived from LCN2 [63]. While native LCN2 does not alter Aβ aggregation, both native L-PGDS and the Anticalins inhibit Aβ aggregation*.* Like ApoD, their effects take place at sub-stoichiometric protein/Aβ ratios. Additionally, L-PGDS has been reported to possess disaggregating capacity [30], while Anticalin H1GA [83] and ApoD (this work) do not share this activity. This functional comparison suggests that ApoD may retain an ancestral function in interacting with amyloid-like molecules, and L-PGDS, an evolutionarily more recent Lipocalin [84], expanded its function by acquiring disaggregating Aβ capabilities. This function was not conserved in LCN2, also recently evolved. Curiously, the selection process for Anticalins generation favored mutations that creates a binding pocket similar to those of ApoD and L-PGDS. The promising finding is that Anticalin H1GA can suppress early synaptic defects in a mouse model of β-amyloidosis when applied at stages preceding plaque formation [83].

In summary, our findings refute the null hypothesis of ApoD being passively trapped in amyloid plaques, and place ApoD as a key extracellular factor actively binding Aβ and modulating Aβ aggregation kinetics through conformation-dependent direct interactions. These findings expand our view of the core biochemical function proposed for ApoD: in addition to managing lipids in cellular membranes and extracellular structures, it is able to manage hydrophobic peptides and condition their dynamics in the extracellular milieu. Based on this new experimental evidence, we can envision ApoD as an endogenous mechanism, naturally turned on in the aging brain, with the ability to chaperon Aβ peptides from its origin in endolysosomal membranes to the formation of plaques. ApoD can act through different phases of the aggregation process, lowering the probability of Aβ monomer nucleation, and slowing down fibril formation and elongation. Eventually, ApoD and Aβ peptides are found together in the less damaging storage place: the amyloid plaques.

## Supplementary Information

Below is the link to the electronic supplementary material.

Supplementary Figures (DOCX 3344 KB)

Supplementary Video S8 (MP4 14343 KB)

Supplementary file S2 (PDB 213 KB)

## Data Availability

All data generated and analyzed during this study are included in this published article and its Online Resources files.

## Abbreviations

ApoD: Apolipoprotein D
Aβ: Amyloid-β
AD: Alzheimer’s disease
APP: Amyloid protein precursor
PBS: Sodium phosphate buffer
ThT: Thioflavin T
Trp: Tryptophan
MD: Molecular dynamics
BCF: Breast cystic fluid
Kd: Dissociation constant
GO: Gene ontology
