# Supplementary material for: Apolipoprotein D acts as a chaperone of Aβ peptide delaying its assembly into amyloid aggregates
Source: Cell Mol Life Sci. 2026 Jul 9;83(1):357. doi: 10.1007/s00018-026-06339-2 (PMC13635072; doi:10.1007/s00018-026-06339-2)
Supplement: Supplementary file 1 [file 18_2026_6339_MOESM1_ESM.docx]

***Fig. S1. Optimization of experimental conditions for accelerated aggregation endpoint assays.***

***(a)*** *Representative time course of Aβ_1-42_ (10 µM) aggregation measured by ThT fluorescence at RT with agitation. ThT alone did not change its fluorescence through the experiment.* ***(b)*** *Controls for each of element involved in the aggregation assay. Only Aβ increases ThT fluorescence (endpoint assay, 3 h at RT with agitation).* ***(c)*** *Characterization of ThT assay dependence on Aβ_1-42_ concentration. Aβ_1-42_ 1 µM was selected as the assay lower limit for detection.* ***(d)*** *Temperature effects on Aβ_1-42_ aggregation at different concentrations. The 1 µM Aβ_1-42_ condition was chosen to test the effect of ApoD^BCF^ (shown in Fig. 2a). ThT fluorescence in C and D was normalized as percentage to control. Box plots show median, quartiles 1 and 3 and minimum and maximum values excluding outliers from n = 3-9 independent experiments. Asterisks point to significant differences in relevant pairs (b, d) or with respect to control (c) after evaluation by ANOVA followed by Holm-Sidak pairwise comparisons. ***p < 0.001.*

***Fig. S3. Best solution from AlphaFold3 (Deepmind) and Protenix docking of Aβ_1-42_ to ApoD.***

***(a)*** *Cartoon of the complex between ApoD (gray) and Aβ_1-42_ (red) generated as best solution by AlphaFold3. The loop between α-helices in this conformation of Aβ interacts with the ApoD binding pocket.* ***(b)*** *Cartoon of the best solution generated by Protenix. In this model the C-terminus stretch of Aβ_1-42_ inserts into ApoD binding pocket. For both models ApoD is shown rotated (upper right corner) to visualize the binding pocket from above. The lower panels show ApoD in this orientation and Aβ further rotated to display their own interacting surfaces, colored by either Kyte-Doolittle’s hydropathy scale (brown for hydrophobic residues, cyan for hydrophilic) or by Coulomb potential (red for negative patches, blue for positive).*

***Fig. S4. Models for ApoD Trp fluorescence upon binding to Aβ in different forms.***

*Trp fluorescence changes plotted against Aβ/ApoD Molar ratios are fitted to the sum of two 3-parameter Hill equations, with Kd (apparent dissociation constant), Fmax (maximum fluorescence change), and b (degree of cooperativity) as the three parameters to be estimated by a least-squares method. Both components (red: fluorescence quenching; blue: provoking fluorescence increase) are shown together with the sum (green). Kd and b values shown for each component.* ***(a-c)*** *Models upon binding of ApoD to Aβ _1-42_ (a), Aβ_1-28_ (b), or Aβ_33-42_ (c) monomers.* ***(d)*** *Model upon binding of ApoD to pre-aggregated Aβ_1-42_ (spontaneous aggregation for 10 days at 4ºC).*

***Fig. S5. Classical Molecular Dynamics of Aβ_1-42_ models predominantly α-helical structures.***

***(a)***  *Secondary structure timeline calculated for the Aβ_1-42_ peptide along a 2.3 µs classical MD computation, together with recorded gyration radii (R_G_). Two major α-helices separated by a loop visibly predominate. The radius of gyration (R_G_) displays strong variations of peptide compactness along the trajectory, like in the 10 µs GA-MD trajectory (Fig. 5).* ***(b)*** *Per-residue frequencies of the various secondary structure elements calculated along the trajectory. General view in upper panel. Enlarged view of extended and β bridge structures in lower panel.* ***(c)*** *Cluster analysis of the trajectory.*

***Fig. S6. Top five AlphaFold3 docking models of the ApoD–Aβ42(K28A) complex.*** *ApoD is shown in blue, with Trp127 highlighted and displayed with all atom structure. The Aβ42(K28A) peptide is shown in red, with the mutated residue Ala28 highlighted in dark blue. In all models, the C‑terminal region of Aβ42 is consistently inserted into the ApoD pocket, whereas no interaction involving the region around residue 28 is observed.*

***Fig. S7.*** ***Hex docking complexes between ApoD and cluster representatives obtained from MD computations at 1/1 and 1/11 ApoD/ Aβ_1-42_ ratios.***

***(a)*** *Complexes obtained by 1/1 ApoD/Aβ ratio rigid docking using Hex software and Aβ conformers representative of 7 highly populated clusters form MD computations. Each panel displays the best docking solutions for each Aβ representative. ApoD molecular surface is in brown, displaying the Lipocalin ligand-binding pocket to the front, and Aβ_1-42_ conformers are shown in colored cartoons.* ***(b)*** *Snapshot at 500 ns from MD computations at 1/11 ApoD/Aβ ratio. ApoD is shown in gray, and its Trp sidechains in light green. Only Aβ_1-42_ molecules bound to ApoD at this time point are shown. The one in cyan is stably bound to the rim of ApoD binding pocket (see supplementary Video S8).*

***Fig. S9. Trajectories of eleven Aβ_1-42_ monomers when modeled in a box with or without a single ApoD molecule.***

***(a)*** *Secondary structure timeline of five examples of Aβ_1-42_ (out of eleven in the box) in the absence of ApoD.* ***(b)*** *Examples of timelines obtained when one molecule of ApoD is present in the box. Extended and β secondary structures, mostly concentrated at the C-terminal portion of the peptide, are more probable to be found in the absence of ApoD (see Fig. 6c for quantitative analysis).*
